# Supplementary material for: Rocket-miR, a translational launchpad for miRNA-based antimicrobial drug development
Source: mSystems. 2023 Nov 17;8(6):e00653-23. doi: 10.1128/msystems.00653-23 (PMC10734502; doi:10.1128/msystems.00653-23)
Supplement: Supplemental figures — Fig. S1 to S5. [file msystems.00653-23-s0001.pdf]

Rocket-miR, a Translational Launchpad for miRNA-based Antimicrobial Drug Development

Supplementary Information

Contents

Figure S1. Zoomed in image of figure 1 (application workflow), panel 1 (summary view)
Figure S2. Zoomed in image of figure 1 (application workflow), panel 2 (miRNA view)
Figure S3. Zoomed in image of figure 1 (application workflow), panel 3 (pathway view)
Figure S4. Zoomed in image of figure 1 (application workflow), panel 4 (compare species view)
Figure S5. Zoomed in image of figure 1 (application workflow), panel 5 (structural analysis view)

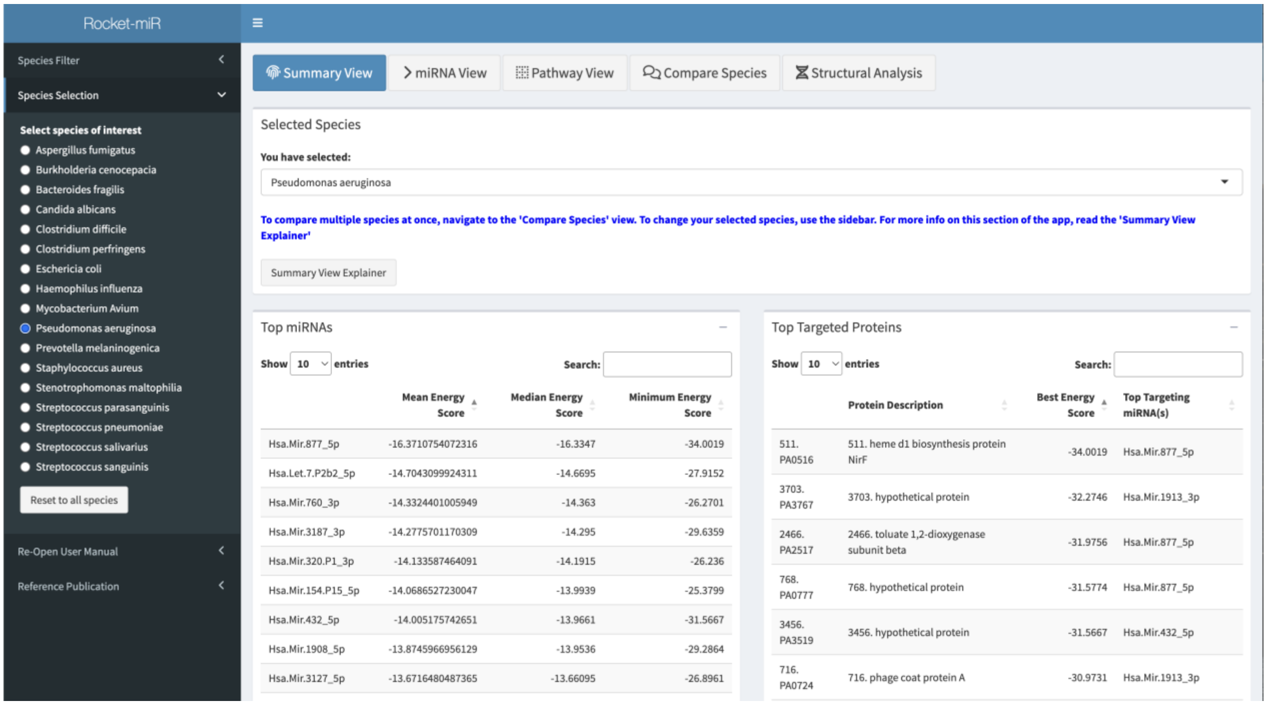

Figure S1. Zoomed in image of figure 1 (application workflow), panel 1 (summary view)

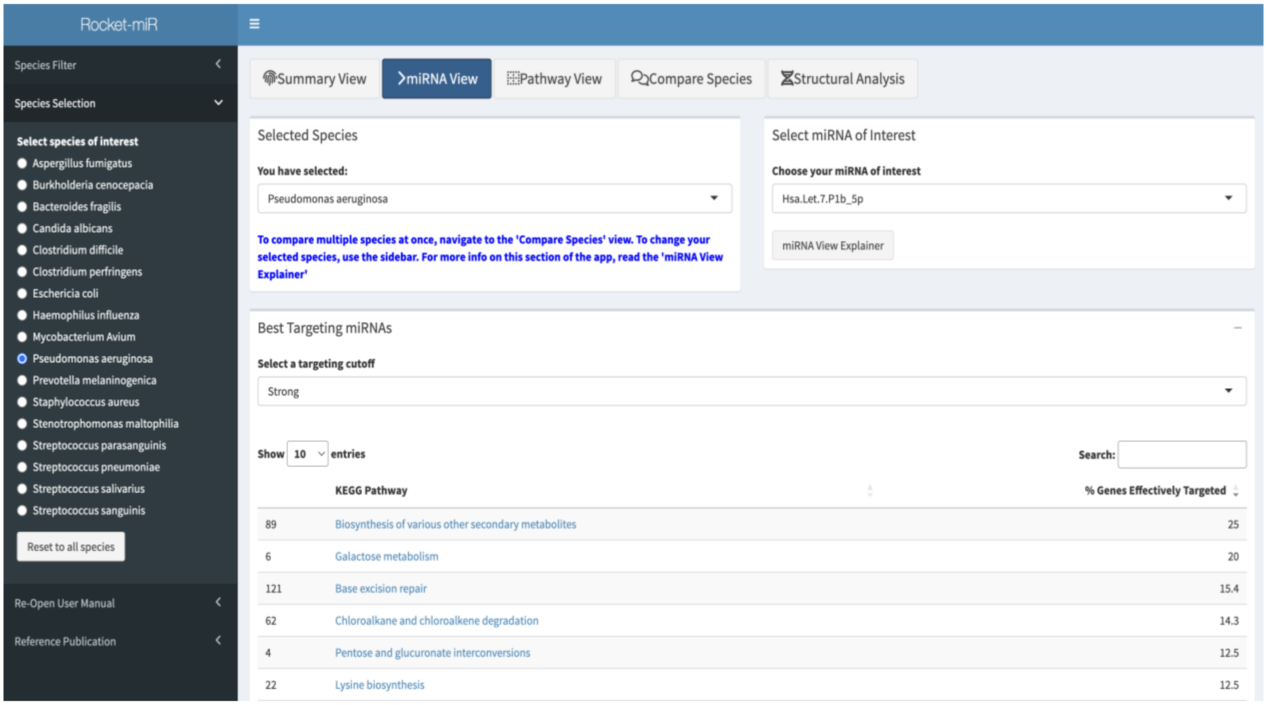

Figure S2. Zoomed in image of figure 1 (application workflow), panel 2 (miRNA view)

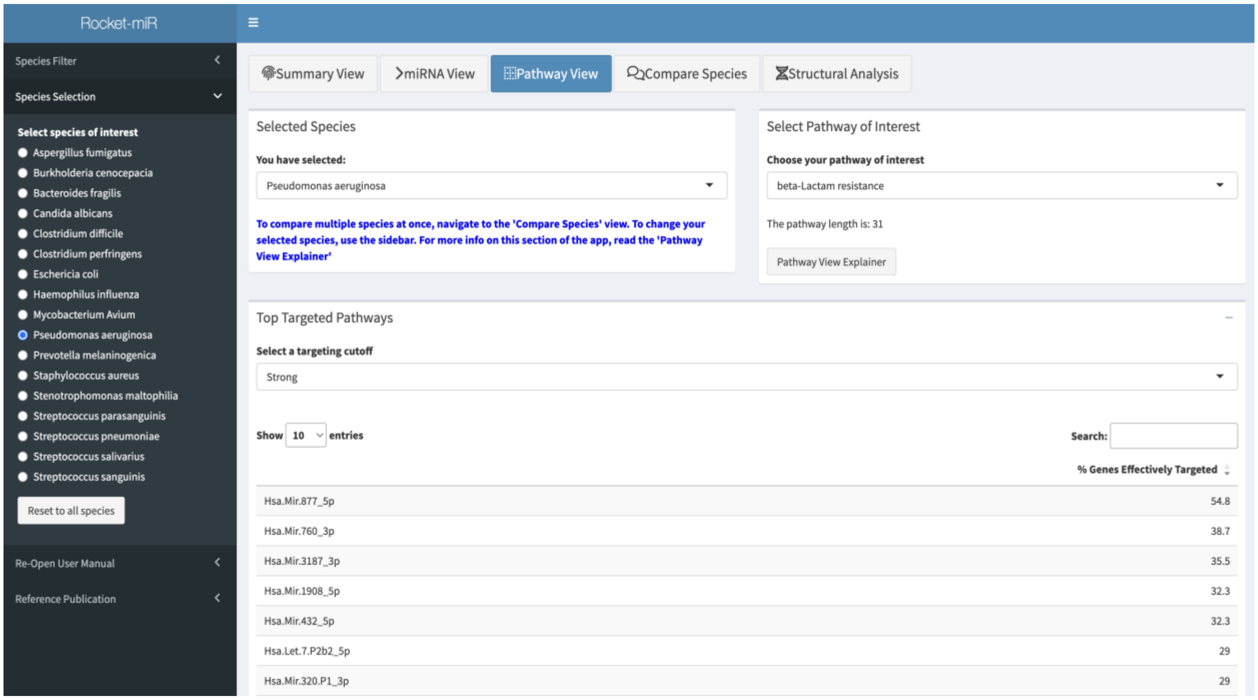

Figure S3. Zoomed in image of figure 1 (application workflow), panel 3 (pathway view)

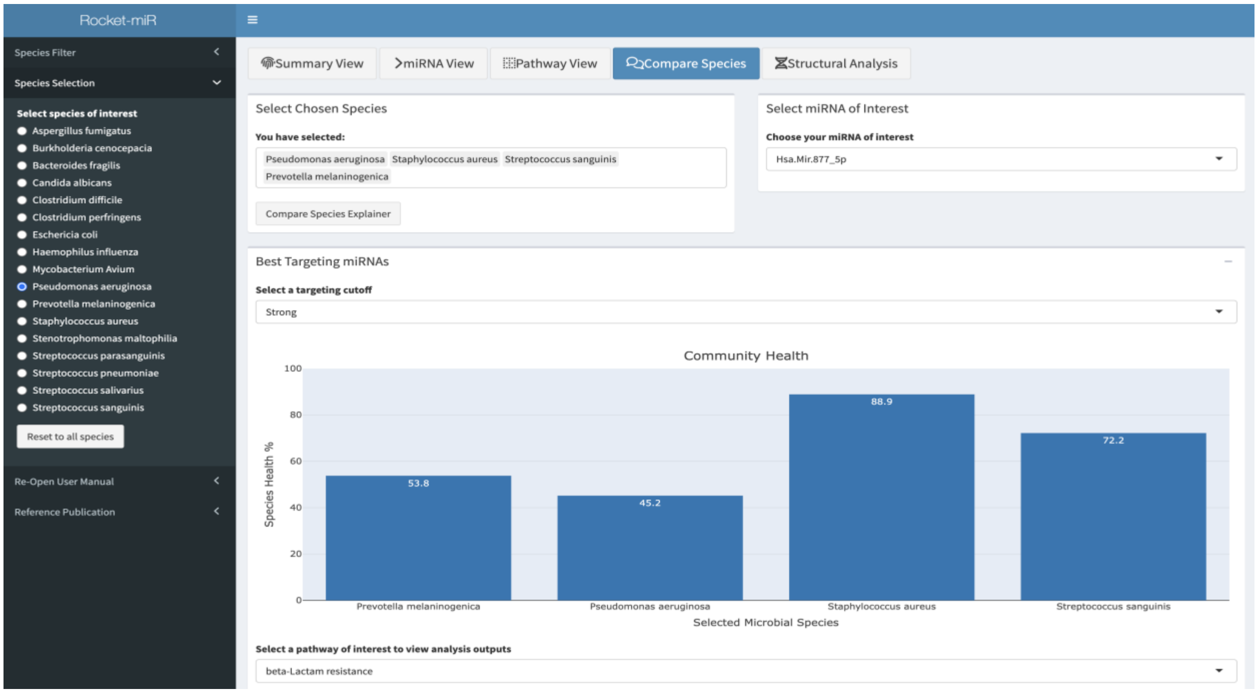

Figure S4. Zoomed in image of figure 1 (application workflow), panel 4 (compare species view)

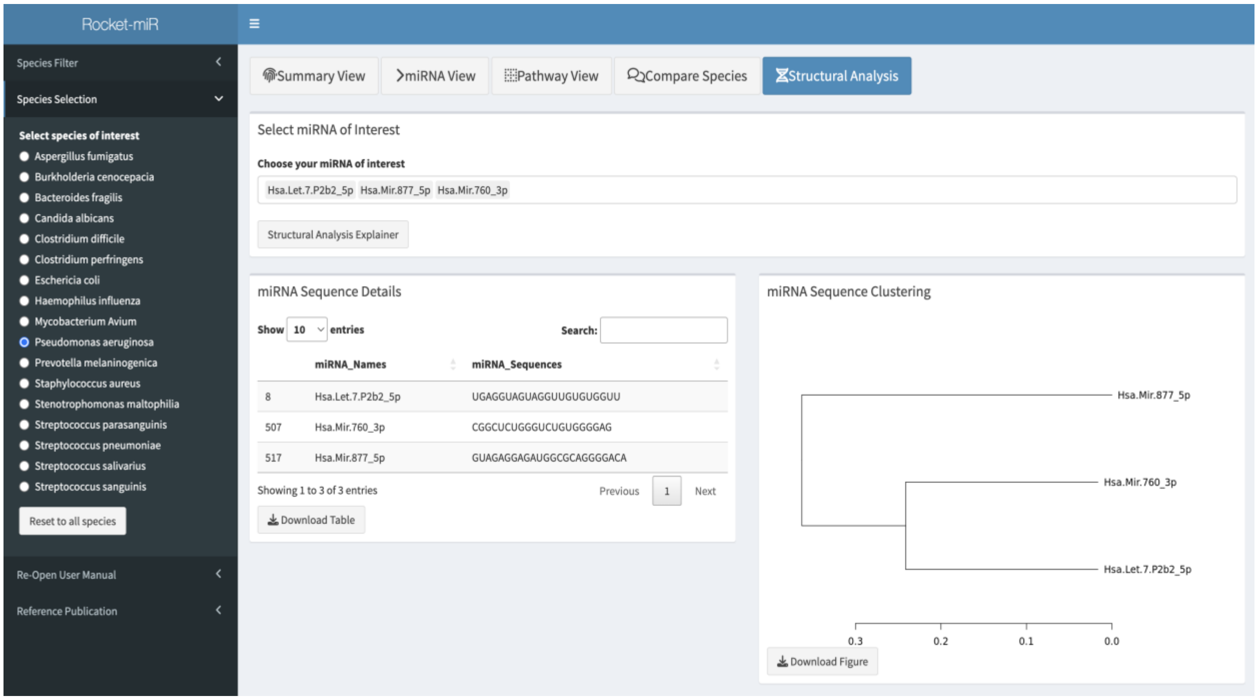

Figure S5. Zoomed in image of figure 1 (application workflow), panel 5 (structural analysis view)
